# Anthropometric Indices and Some Aspects of Physical Fitness in Croatian Adolescents by Gender

**DOI:** 10.3390/ijerph16142582

**Published:** 2019-07-19

**Authors:** Martin Zvonar, Mario Kasović, Lovro Štefan

**Affiliations:** 1 Faculty of Sport Studies, Masaryk University, 625 00 Brno, Czech Republic; 2 Department of General and Applied Kinesiology, Faculty of Kinesiology, University of Zagreb, Horvaćanski zavoj 15, 10 000 Zagreb, Croatia

**Keywords:** secondary-school students, body-mass index, waist circumference, fitness

## Abstract

*Background*. The main purpose of this study was to explore the body-mass index and waist circumference associated with physical fitness by gender. *Methods.* In this cross-sectional study, we included 1036 adolescents (55.4% girls) from eight randomly selected secondary schools within the city of Zagreb (Croatia). Body-mass index and waist circumference were objectively measured. Physical fitness included three tests: (1) 1 min sit-ups, (2) standing long jump and (3) a sit-and-reach test. Associations were calculated using linear regression models. *Results.* Boys had higher body-mass index and waist circumference values, compared to girls (*p* < 0.001). They also performed better in 1 min sit-ups and the standing long jump tests (*p* < 0.001), while girls obtained higher values in the sit-and-reach test (*p* < 0.001). In boys, body-mass index and waist circumference were associated with 1 min sit-ups and the standing long jump. In girls, waist circumference was also associated with 1 min sit-ups and the standing long jump, while body-mass index was only associated with this standing long jump. *Conclusions.* Our study shows that anthropometric indices have non-linear associations with physical fitness tests in a large sample of Croatian adolescents. Screening for thinness and obesity to predict the level of physical fitness should be of a great interest.

## 1. Introduction

Obesity has become a major public health problem in the past three decades, especially in children and adolescents [1]. Specifically, the prevalence of obesity in this age group in 2016 was 5.6% in girls and 7.8% in boys [2]. In Croatia, the data from the Health Behavior in School Aged Children 2009/2010 survey [3] showed the prevalence of those overweight being 23.0% and 10.0% in boys and girls, respectively. Obesity is associated with many non-communicable diseases [4], and higher levels of excessive fat in childhood and adolescence lead to negative health consequences in adulthood [2]. Similar to being overweight or suffering from obesity at one end, thinness also represents a potential public health problem at the other end, and is associated with higher rates of mortality and several diseases [5,6].

Physical fitness represents a significant part of physical activity [7]. It is defined as ‘a measure of the capacity to perform physical activity and/or physical exercise that integrates the majority of the bodily functions involved in body movement’ [7]. Similarly just like obesity, the level of physical fitness in childhood/adolescence often persists later on in adulthood [8]. Moreover, it has been shown that higher levels of physical fitness in children and adolescents lead to better overall health later on in life [9].

Several previous studies have examined the association between overweight/obesity (by using the body-mass index indicator) and physical fitness [10,11,12,13,14,15]. Of those, only a few have calculated a quadratic association including both thinness and obesity in the equation [13,15,16,17].

Results of those studies were consistent with each other, showing a non-linear association between the body-mass index and several fitness tests; that is both thin and overweight/obese participants scored lower on the physical fitness tests. According to the aforementioned, more studies have been using the body-mass index as an indicator of nutritional status, yet the body-mass index cannot differ fat mass from free-fat mass, and may lead to a misclassification [18]. Thus, some other indicators of obesity, like waist circumference, have been proposed [19]. Fewer studies have examined the association between waist circumference [11,12,14] and physical fitness.

After an extensive literature review, there has been a lack of studies examining combined non-linear associations between body-mass index and waist circumference with physical fitness. Therefore, the main purpose of the study was to explore the body-mass index and the waist circumference associated with physical fitness in a large sample of Croatian adolescents aged 15–18 years by gender. We hypothesized that the association between all anthropometric indices and physical fitness would be non-linear with a parabolic shape.

## 2. Materials & Methods

### 2.1. Participants

In this cross-sectional study, participants were secondary-school students. A detailed study protocol has been described elsewhere [20]. In brief, we randomly selected 11 (8 grammar and 3 vocational) out of 86 secondary-schools in the city of Zagreb, after which we randomly selected one class representing each grade within the school (from 1st to 4th). Each class had ≈25 students. All students were considered healthy, and were not affected by diseases. The selection criteria were: (1) An active participation in physical education classes and (2) an absence of injuries. According to the Croatian Bureau of Statistics for the year 2017 [21], there were 36,350 secondary-school students in total. Our sample size was estimated to be 1030, by using a 95% confidence level and a 3% margin of error. All procedures performed in this study were in accordance with the Declaration of Helsinki, and were approved by the Institutional Review Board of the leading author (code: 02/2019). Also, all of the participants and their parents/guardians provided written informed consent for participation in the study.

### 2.2. Anthropometric Measures

Body height was measured to the nearest millimeter in bare or stocking feet with the adolescent standing upright against a stadiometer (Seca, Japan). The result was given in meters. Body weight was measured to the nearest 0.1 kg, and the participant wore light clothes with no shoes (Seca, Japan). The result was given in kilograms. BMI (kg/m^2^) was calculated as weight (in kilograms) divided by the square of height (in meters). WC was measured for each participant remaining still in a standing position. We used anthropometric tape placed horizontally midway between the lower rib margin and the iliac crest at the end of normal expiration [22]. The association between BMI and WC was 0.84 in boys and 0.73 in girls (*p* < 0.001).

### 2.3. Objectively Estimated Physical Fitness

We used a EUROFIT Battery Fitness Test to assess the level of physical fitness in adolescents. These tests are considered reliable and valid instruments to measure the level of physical fitness in children and adolescents [23]. Standing long jump, sit-ups for 1 min and a sit-and-reach test were chosen because of their mutual independence to the other [24]. Data were collected by two trained researchers in order to guarantee the standard measurement methodology [24]. A brief explanation of each test is presented below:

Standing long jump: Each subject performed distance jumps from a standing start. While performing the jumps, the subjects were asked to bend their knees with their arms in front of them, parallel to the ground, then to swing both arms, push off vigorously and jump forward as far as possible, trying to land with their feet together and to stay upright. The best out of two attempts was taken as the final score (expressed in centimeters) [25].

Sit-ups in 1 min: Trunk strength was assessed as the maximum number of sit-ups achieved in one minute. Children were seated on the floor, backs straight, hands clasped behind their neck, knees bent at 90°, with their heels and feet flat on the mat. They then lay down on their backs, shoulders touching the mat, and returned to the sitting position with their elbows out in front to touch their knees, keeping the hands clasped behind their neck the whole time. The total amount of correctly performed sit-ups in 60 s was the score [25].

Sit-and reach test: Sitting on the floor or upon a mat, legs straight under the angle of 90°, the person being tested reached forward with the arms (hands overlapping). The distance of reach was measured in centimeters using a measuring non-elastic tape attached to the floor [26].

### 2.4. Data Analysis

Data are presented as mean (SD). All variables were grouped according to sex (boys vs. girls). First, we calculated the differences between the sexes in variables which were analyzed using univariate analysis of variance. Previous studies have shown sex differences in perceived and estimated physical fitness [27]. Therefore, we presented sex-stratified analyses. Next, we calculated quadratic associations between the anthropometric indices and each physical fitness test, using linear regression analyses. Finally, we also checked for multicollinearity between these physical fitness tests using the variance inflation factor (VIF). The VIF value was <2, indicating no multicollinearity between the physical fitness tests. Significance was set up at *p* ≤ 0.05, and it was two sided (2-sided). All of the analysis were performed in the Statistical Package for Social Sciences Software, ver. 22 (IBM Corp., Armonk, NY, USA).

## 3. Results

Basic descriptive statistics of the study participants are presented in Table 1. Boys had higher body-mass index (Effect size (ES) = 0.66) and waist circumference (ES = 0.93) values, compared to girls (*p* < 0.001). They also performed better in 1 min sit-ups (ES = 0.81) and standing long jump (ES = 1.69) tests (*p* < 0.001), while girls obtained higher values in the sit-and-reach test (ES = 0.73, *p* < 0.001).

**Figure 1:**
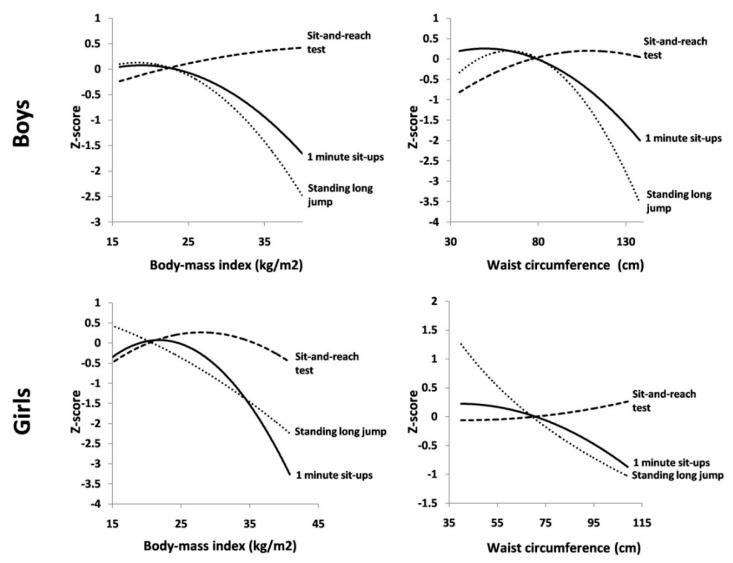
Quadratic associations between anthropometric indices and physical fitness in boys and girls, Croatia (2019).

Quadratic associations between anthropometric indices and physical fitness tests and overall physical fitness for both boys and girls are presented in Table 2 with a graphical presentation in Figure 1. Among boys, a quadratic regression showed that their body-mass index was associated with 1 min sit-ups and a standing long jump, while no significant association with the sit-and-reach test was observed. Waist circumference was also only associated with 1 min sit-ups and standing long jump. In girls, their waist circumference was associated with 1 min sit-ups and the standing long jump, while the body-mass index was only inversely associated with the standing long jump.

## 4. Discussion

The main question of the study was as to whether body-mass index and waist circumference were associated with some aspects of physical fitness. This study shows that both body-mass index and waist circumference are associated with physical fitness tests, especially 1-min sit-ups and the standing long jump.

Our results of the association between body-mass index and physical fitness are in line with previous studies conducted among a sample of children [13,15,17] and adolescents [13,15,16]. Most recently, Lopes et al. [16] showed a non-linear (curvilinear) association between body-mass index and standing long jump among boys aged 10–11 and 14–17 years and girls aged 10–13 years, while among girls aged 14–17 years, the association was linear. The same group of authors also found a non-linear association between body-mass index and push-ups and a multistage shuttle run in both boys and girls [16]. A study conducted among Croatian untrained boys aged 14–16 years showed a non-linear association between several anthropometric and physical fitness variables [17]. Although previous studies have found a decline in fitness or motor performance with higher body-mass index values [28], a study by Chen et al. [27] found that lower levels of body-mass index were associated with a better aerobic capacity in Taiwanese children aged 7–8 years, pointing out that different ranges of body-mass index are not similarly associated with physical fitness tests.

Our study also shows a non-linear association between waist circumference and standing long jump and overall physical fitness score in boys, and overall physical fitness score in girls. Only a few previous studies have examined the aforementioned association [11,12,14]. However, none of those studies used a quadratic analysis to calculate the association between waist circumference and physical fitness. Nevertheless, similar results were observed showing an inverse association between waist circumference and physical fitness components [11,12,14]. Similar to body-mass index, a higher level of waist circumference often means excess body fat acting like an extra load during physical fitness test performances [12]. Also, obese individuals tend to have a lack of motor learning and reduced activation in motor units, which may explain poorer lower extremity strength [29].

### 4.1. Study Strengths

This study has several strengths. First, we conducted our study on a relatively large sample of secondary-school students (*N* = 1036). Second, we used both body-mass index and waist circumference as indicators of obesity. Third, we presented non-linear regression results, regarding anthropometric indices and some aspects of physical fitness.

### 4.2. Study Limitations

Our study has several limitations. First, by using a cross-sectional design, we cannot conclude the causality of the correlation. Second, to assess objectively estimated physical fitness, we only used musculoskeletal fitness. Previous studies have used aerobic capacity as the measure of physical fitness [10,11,12,13,14,15,16,17]. However, a most recent meta-analysis of longitudinal studies has revealed a moderate-large negative association between muscular fitness in childhood/adolescence and adiposity and cardiometabolic parameters in adulthood, pointing out that muscle-strengthening activities have beneficial effects on health during lifespan and might be of greater importance than aerobic capacity [30]. Third, we did not collect information about biological maturity status, since individual specific height and weight growth might have led to different associations. Finally, although we conducted the study on a large sample of urban secondary-school students, and the data cannot be used for a rural or mixed population, our results can be used for comparisons in adolescents with different nationalities.

## 5. Conclusions

Our study shows that anthropometric indices are associated with some aspects of physical fitness tests in a large sample of Croatian adolescents. Although overweight and obesity have become a major public health problem worldwide, our study also shows that thin adolescents might also be a group at risk of performing poorly on physical fitness tests. Therefore, screening for both thinness and obesity to predict the level of physical fitness should be of great interest for future longitudinal research, and those findings might serve as certain recommendations for optimal nutritional status and better physical fitness performance in adolescents.

## Figures and Tables

**Table 1:** Basic descriptive statistics of the study participants, (Croatia, 2019).

Study Variables	Total Sample	Boys	Girls	*p*-Value
	(*N* = 1036)	(*N* = 463)	(*N* = 573)	
	Mean (SD)	Mean (SD)	Mean (SD)	
	Min-Max	Min-Max	Min-Max	
Age (yrs)	16.3 (1.1)	16.4 (1.1)	16.2 (1.1)	<0.001
	15–18	15–18	15–18	
Anthropometric Indices				
Body-mass index (kg/m^2^)	21.3 (2.9)	22.0 (3.2)	20.1 (2.6)	<0.001
	14.3–40.9	15.9–40.9	14.3–40.8	
Waist circumference (cm)	73.3 (9.4)	77.6 (9.6)	69.7 (7.5)	<0.001
	35.0–138.0	35.0–138.0	40.0–109.0	
Physical Fitness				
1 min sit-ups (#)	51.2 (11.7)	56.1 (11.9)	47.3 (10.0)	<0.001
	20.0–112.0	20.0–112.0	20.0–82.0	
Standing long jump (cm)	186.1 (32.9)	209.6 (29.2)	167.1 (21.4)	<0.001
	72.0–280.0	102.0–280.0	72.0–225.0	
Sit-and-reach test (cm)	67.1 (13.5)	62.0 (12.0)	71.2 (13.2)	<0.001
	15.0–112.0	25.0–112.0	15.0–105.0	

**Table 2:** Quadratic regression associations between anthropometric indices and physical fitness tests in boys and girls, Croatia (2019).

Study Variables	Boys (*N* = 463)			Girls (*N* = 573)		
	1 min sit-ups (#)	Standing long jump (cm)	Sit-and-reach test (cm)	1 min sit-ups (#)	Standing long jump (cm)	Sit-and-reach test (cm)
Anthropometric Indices	Standardized β coefficient (*p*-value)	Standardized β coefficient (*p*-value)	Standardized β coefficient (*p*-value)	Standardized β coefficient (*p*-value)	Standardized β coefficient (*p*-value)	Standardized β coefficient (*p*-value)
Body-mass index (kg/m^2^)	−0.62 (0.047)	−0.89 (0.004)	−0.12 (0.701)	−0.69 (<0.001)	−0.18 (0.530)	−0.54 (0.063)
Waist circumference (cm)	−0.30 (<0.001)	−0.42 (<0.001)	−0.04 (0.635)	−0,20 (<0.001)	−0.24 (<0.001)	−0.13 (0.032)

*p* < 0.05.
